# Synthesis of Pd–Ru solid-solution nanoparticles by pulsed plasma in liquid method

**DOI:** 10.1039/c9ra10003b

**Published:** 2020-04-01

**Authors:** Tsutomu Mashimo, Shota Tamura, Kenta Yamamoto, Zhazgul Kelgenbaeva, Weijan Ma, Makoto Tokuda, Michio Koinuma, Hiroshi Isobe, Akira Yoshiasa

**Affiliations:** Institute of Pulsed Power Science, Kumamoto University Kumamoto 860-0862 Japan mashimo@gpo.kumamoto-u.ac.jp; Faculty of Engineering, Kumamoto University Kumamoto 860-0862 Japan; Faculty of Science, Kumamoto University Kumamoto 860-0862 Japan

## Abstract

We have synthesized solid-solution nanoparticles (Pd : Ru = 1 : 3, 1 : 1 and 3 : 1) in an immiscible Pd–Ru system by the pulsed plasma in liquid method using Pd–Ru mixture bulk electrodes. The particle sizes of the floated and sedimented samples were measured to be <10 and <20 nm, respectively, *via* high-resolution transmission electron microscopy (HR-TEM). The lattice parameters of nanoparticles followed the Vegard's law, and the energy-dispersive X-ray spectroscopy (EDX) results almost coincided with those obtained for the starting bulk mixtures. The solid-solution structures and local structure were confirmed *via* HR-TEM, X-ray photoelectron spectroscopy (XPS) and X-ray absorption fine structure spectroscopy (XAFS).

## Abstract 2

We have synthesized solid-solution nanoparticles (Pd : Ru = 1 : 3, 1 : 1 and 3 : 1) in an immiscible Pd–Ru system by the pulsed plasma in liquid method using Pd–Ru mixture bulk electrodes.

## Graphical abstract

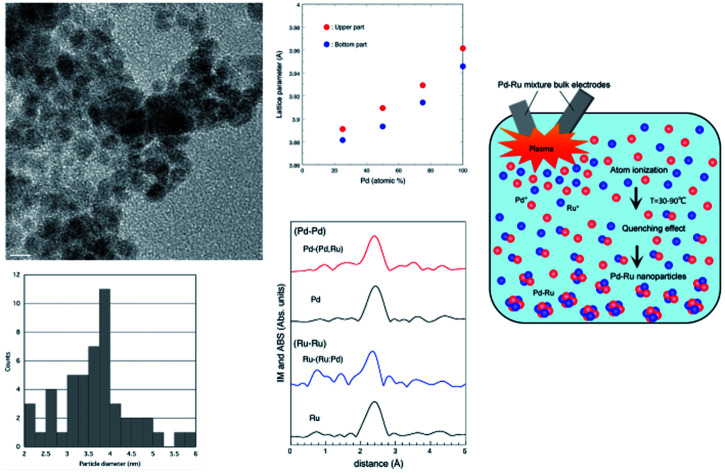

## Introduction

Pt, Pd, Rh and Ru have been used as three-way catalysts, and Pd is well-known as a hydrogen storage metal.^1–4^ Pd, Rh and Ru are neighboring noble elements in the 4d transition metal series. No combination of binary alloy from these elements (Ru–Rh, Rh–Pd and Ru–Pd) forms a solid-solution alloy throughout the whole composition range at room temperature.^5,6^ Rh has high catalyst activity; for example, NOx reduction catalysts,^7,8^ but it is one of the most expensive elements. If Pd–Ru solid-solution nanoparticles (in which Rh exists between Pd and Ru in the periodic table) are synthesized, it is expected that such nanoparticles show excellent catalytic properties, which is equal to or better than Rh due to the interactions of 4d electrons. However, it is not so easy to synthesize the Pd–Ru solid-solution nanoparticles because this system is immiscible. Kusaba *et al.*^9^ reported the synthesis of Pd–Ru solid-solution nanoparticles *via* a chemical reduction method.

Our previous studies have shown that the pulsed plasma in liquid (PPL) method^10^ is a good one-step method for synthesizing numerous types of nanomaterials. This process is relatively cheap and environmentally friendly. The short duration (several microseconds) and the quenching effects of the surrounding cold liquid limit the size of the crystals, which enables the synthesis of very small and/or metastable particles. We have synthesized the nanoparticles of such materials as single-element metals,^11^*etc.* carbon-coated metals,^12^ nano-graphene^13^ and compound nanoparticles^14^*etc.* Recently, we synthesized Fe–Pd and Ag–Cu alloy nanoparticles in the half miscible and immiscible system, respectively, at room temperature.^15,16^

In this study, we seek to synthesize solid-solution nanoparticles in the immiscible Pd–Ru system at room temperature *via* a one-step physical method, *i.e.* the PPL method, using the Pd–Ru bulk mixture electrodes of the same composition. We expected the synthesis of an excellent three-way catalyst, hydrogen reduction catalyst, *etc.*, *via* a simple, nontoxic, and low-cost technique compared to conventional techniques.

## Experimental

**Fig. 1:**
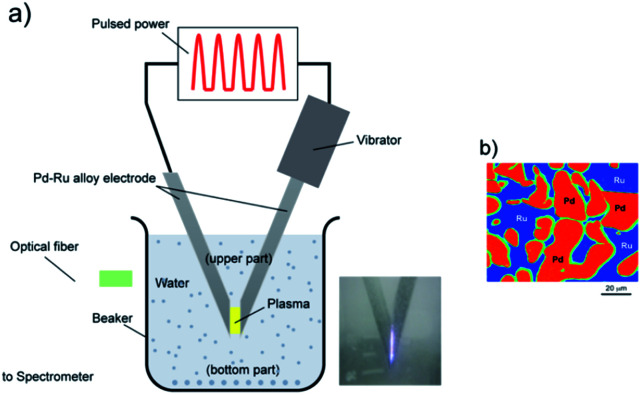
Experimental set up of pulsed plasma in liquid method for the synthesis of Pd–Ru solid-solution nanoparticles (a), and the EPMA photo of the Pd–Ru (1 : 1) mixture bulk electrode (b).

A schematic of the experimental setup is shown in Fig. 1a. Metal electrodes (cathodes and anodes) were submerged in a liquid connected to a power source.^10^ The impulse plasma was produced by the spark discharge between two electrodes. The gap between the two electrodes was approximately 1 mm, and the electrical current pulses with about<20 μs duration. One of the electrodes was kept vibrating, so that the discharging process could proceed continuously.

In this study, we used pure Pd and Ru bulk electrodes and the Pd–Ru mixture bulk electrodes, each with the Pd–Ru compositions of 1 : 3, 1 : 1 and 3 : 1. The electrodes with 3 mm in diameter and 50 mm in length were prepared by melting a Pd and Ru mixture, whose purity was 99.99%, and provided by the Rare Metallic Co., Ltd. Fig. 1b shows the EPMA photo of an electrode (Pd : Ru = an atomic ratio of 1 : 1), in which the Pd and Ru grains of 10–50 μm in size are observed as a mixture. The Pd–Ru electrodes were first dipped into a 200 mL of 99% ethanol solution and were applied a pulsed voltage of 60.5 V for 60 min. The current range was on an average 1–2 A. After the experiment, the samples were separated from the liquid into floating (upper) and sedimented (bottom) parts using a centrifuge, and then dried. Then, we used an electric stove to dry these two parts for 4 h.

During the synthesis, the atomic emission spectra of the plasma discharge were recorded on an ALS SEC2000 UV-Vis optical spectrometer placed close to the plasma discharge zone outside the quartz beaker. Emission spectrum peaks were identified according to the NIST1 database.^17^

The X-ray diffraction (XRD) patterns for the samples were recorded on a Rigaku RINT-2500 VHF diffractometer, using Cu Kα radiation at 40 kV and 200 mA. We used the high-resolution transmission electron microscopy (HR-TEM) (Philips Tecnai. F20) to observe the morphology and microstructure of the as-prepared samples. An elemental analysis was performed using a JEOL JSM-7600F energy-dispersive X-ray (EDX) spectroscope at 15 kV with a point resolution of 1.0 nm. X-ray photoelectron spectroscopy (XPS) (Sigma Probe, Thermo Scientific, USA) was performed with monochromatic Al Kα radiation from a monochromatic source gun with a spot size of 400 μm and a pass energy of 100 eV.

The X-ray absorption fine structure (XAFS) spectra near the Ru and Pd K-edge were recorded in transmission mode (beam size 1.2 × 0.3 mm) at beamline NW10A AR, KEK, Tsukuba, Japan. Synchrotron radiation was monochromatized *via* a Si (311) double-crystal monochromater. X-ray energy calibration was performed by setting the copper metal pre-edge absorption peak to 8978.8 eV. Mirrors were used to eliminate higher harmonics. The radial structural function was obtained *via* Fourier transform over the *k* range of 2.5 < *k* < 10.5 Å^−1^. We used the Fourier filtering technique and a non-linear least-squares structure parameters fitting method with an analytical EXAFS formula. The single-shell fitting was carried out for the first nearest neighbour distance.

## Results and discussion

### X-ray diffraction (XRD) analysis

**Fig. 2:**
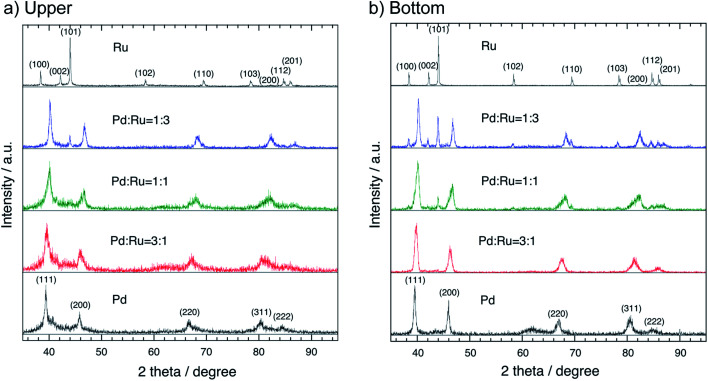
XRD patterns of the upper (a) and bottom (b) parts of the Pd–Ru (1 : 3, 1 : 1 and 3 : 1) alloy nanoparticles, and Pd and Ru element nanoparticles.

Fig. 2 shows the XRD patterns of the floated (upper) (a) and sedimented (bottom) (b) part samples of the Pd–Ru alloy together with those of Pd and Ru single element nanoparticles obtained by the PPL method. The XRD patterns of the 3 : 1 and 1 : 1 nanoparticles show the FCC Pd phase. However, one of the 1 : 3 nanoparticles shows the mixture patterns of the FCC Pd phase and the HCP Ru phase. Each peak of the upper part samples is broader than that of the bottom part samples, which may be caused by the smaller particle size of the upper part samples. The lattice parameters (*a*_0_) of each upper and bottom part samples of the Pd–Ru alloy and Pd single element are summarized in Table 1. The *a*_0_ of Pd nanoparticles (3.9495(17)) is larger than that of the commercial Pd sample (3.8895(14)). This may be due to the larger surface area of the upper nanoparticles. The lattice parameter of the upper sample (3.8998(16) and 3.8988(17) Å) of the 1 : 1. The lattice parameter of the present solid-solution nanoparticles is smaller than that of pure Pd nanoparticles (3.9495(17) Å).

**Table 1:** Lattice parameters

Sample	Upper, (Å)	Bottom, (Å)
Pd : Ru = 3 : 1	3.9353(6)	3.9143(19)
Pd : Ru = 1 : 1	3.9094(9)	3.8934(7)
Pd : Ru = 1 : 3	3.8911(3)	3.8815(4)
Pd single nano	3.9616(7)	3.9458(7)
Pd commercial	3.8895(14)	

**Fig. 3:**
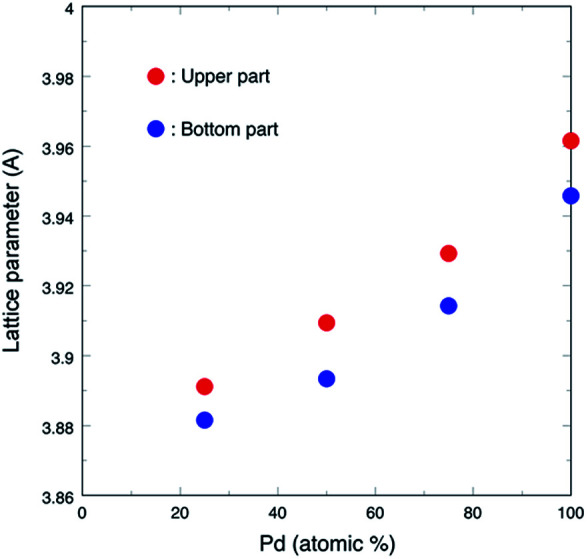
Lattice parameter of the FCC phase *versus* composition.

Fig. 3 shows the lattice parameters *versus* composition calculated using a software named JADE 5.0 of Materials Data Inc. The lattice parameter increased with the Pd ratio, which followed the Vegard's law, thus revealing that the as-synthesized Pd–Ru samples consisted of solid-solution nanoparticles with the FCC structure. The lattice parameters of the upper part samples are larger than those of the bottom ones. This may be due to the larger surface area of nanoparticles.

### High resolution transmission electron microscopy (HRTEM)

**Fig. 4:**
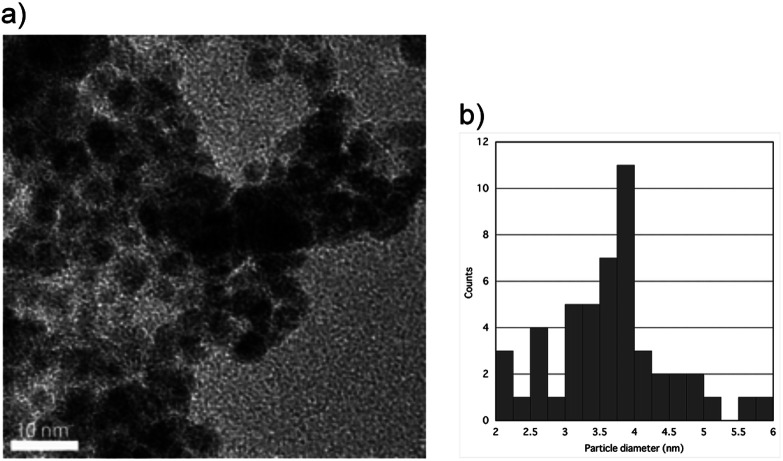
HR-TEM image of the upper sample of the 1 : 1 Pd–Ru alloy nanoparticles (a) and the particle size distributions (b).

**Fig. 5:**
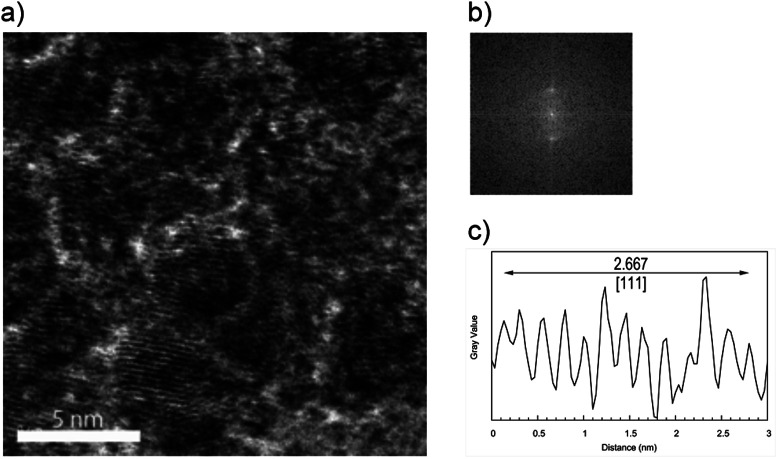
Expanded HR-TEM images of selected areas of upper sample (a), the corresponding fast Fourier transform patter (b) and the gray value plot along the [111] direction (c).

Fig. 4 shows the HRTEM image of the upper sample of the 1 : 1 Pd–Ru alloy nanoparticles (a) with the particle size distributions (b). The image shows that the nanoparticle size was less than 10 nm, while that of bottom sample was less than 20 nm, and this result is consistent with that of the XRD patterns. It can be seen from these figures that the average particle diameter of the upper is less than 4 nm and that of the bottom is less than 6 nm. Fig. 5 shows the expanded HR-TEM images of the selected areas of upper sample (a), corresponding to the fast Fourier transform pattern (b) and gray value plot along the [111] direction with the average values of 2.667 Å (c) (upper). In this figure, we can see lattice ordering on the nano scale.

**Fig. 6:**
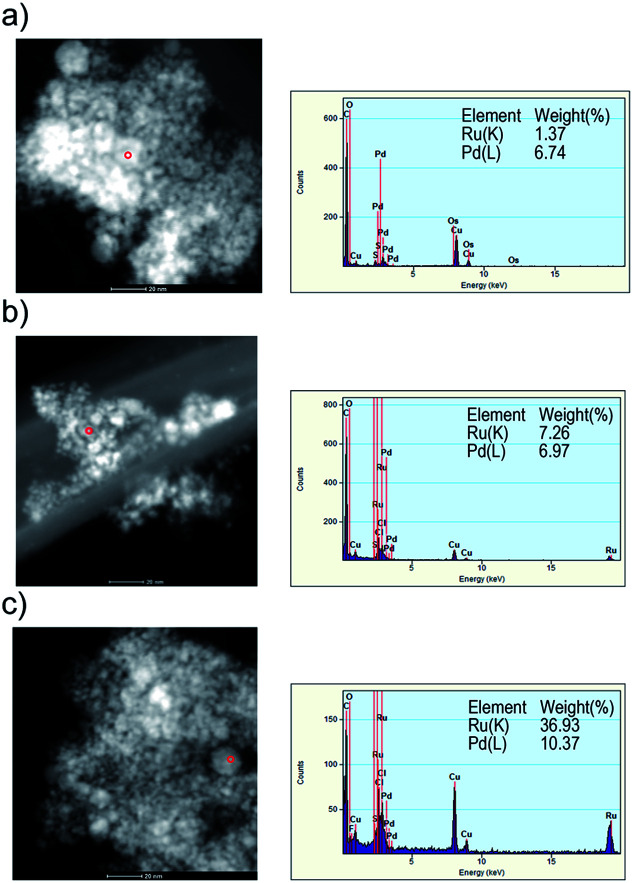
HAADF-STEM image and EDX analysis of the Pd–Ru (1 : 3 (a),1 : 1 (b) and 3 : 1 (c)) alloy nanoparticles.

Fig. 6a–c represent the HRTEM energy dispersive X-ray spectrum (EDX) of the obtained nanoparticles when Pd : Ru = 3 : 1, 1 : 1 and 1 : 3, respectively. The compositions of element were measured at points in particle as red circle of the high-angle annular dark-field scanning transmission electron microscopy (HAADF-STEM) image. The red circle (<10 nm) indicated the measurement area of the EDX. The rates of Pd and Ru element atomic% of the samples obtained by using the alloy electrodes of Pd : Ru = 3 : 1, 1 : 1 and 1 : 3 were 6.7 : 1.4, 7.0 : 7.2 and 1.0 : 3.7, respectively, which is roughly comparable to those of the starting bulk mixture electrode. As a result of the XRD and TEM results, it can be concluded that the Pd–Ru solid-solution nanoparticles with any composition rate can be synthesized *via* the PPL method using the Pd–Ru bulk mixture electrodes.

### X-ray photoelectron spectroscopy analysis (XPS)

**Fig. 7:**
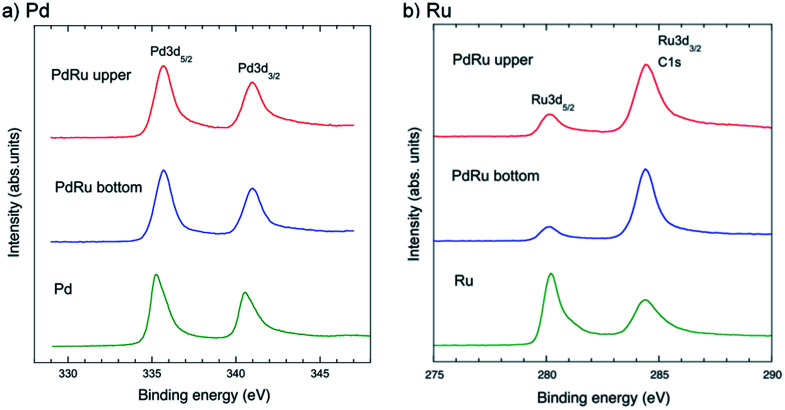
XPS spectra of the upper and bottom parts of the Pd–Ru samples and the refered spectra of Pd (a) and Ru (b) in the range of 275–295 eV and 330–346 eV, respectively.

We studied the change in the chemical composition and valence states of the 1 : 1 Pd–Ru nanoparticles using XPS, a surface-specific technique. Fig. 7a and b show the XPS spectra of the upper and bottom part samples of Pd–Ru and the corresponding spectra of Pd and Ru in the range of 330–346 eV and 275–295 eV, showing the core-level peaks of Ru3d_3/2_ and Pd3d_5/2_, respectively. The Pd binding energy of Pd3d_5/2_ and Pd3d_3/2_ of the Pd–Ru alloy shifted from 335.23 and 340.53 eV to 335.7 and 341.0 eV, respectively, which may show the change in the binding state of Pd–Pd. It is reasonably understood by considering that the Pd 3d electrons of the Pd–Ru alloy have plus charge and are not so easy to jump out from electron orbits. Moreover, the Ru binding energies of Ru3d_5/2_ (280.2 eV) and Ru3d_3/2_ (284.4 eV) of the Pd–Ru alloy were not much different from those of the Ru nanoparticles. However, the peak intensities change because C1s peak (284.8 eV) may overlap at that of the Pd3d_3/2_ peak. It is assumed that the C1s peak of the carbon spectra may have appeared by the carbon impurity from air dust or the double-sided tape used in the measurement.

### X-ray absorption fine structure (XAFS) spectra

**Fig. 8:**
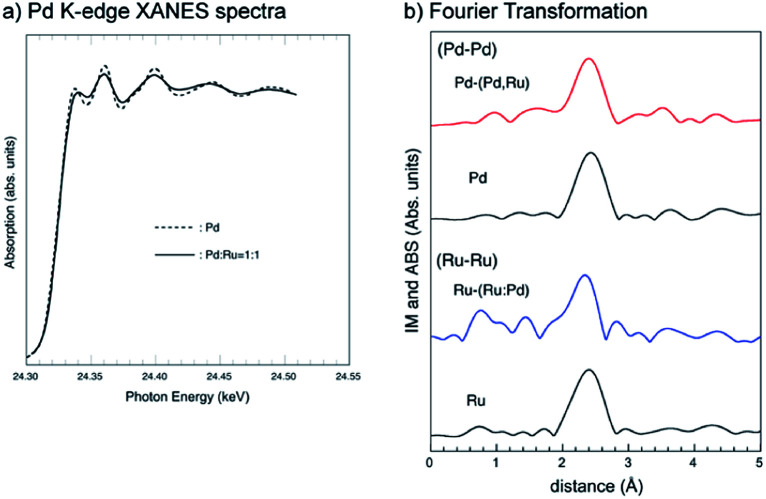
Experimental Pd K-edge XANES spectra for the bottom parts of Pd and Pd–Ru samples (a), and the Fourier transform of the Pd and Ru K-edge EXAFS oscillation function *k*^3^*χ*(*k*) of Pd–Ru, Pd and Ru samples (b). No phase shift correction was applied.

The Pd K-edge XANES spectra for the bottom parts of the 1 : 1 Pd–Ru nano particles are shown in Fig. 8a. The Pd K XANES spectrum of the Pd–Ru sample is similar to that of Pd sample. The amplitude of the XAFS signal for the Pd–Ru sample is more damped than that for Pd sample. Damping is caused by an increase in the Debye–Waller factor for the XAFS signal due to the statistical arrangement of different elements with different sizes and electronic structures. This damping indicates solid-solution formation with the increase in the local statistical configuration.

Fig. 8b shows the Fourier transform (distribution function) of the Pd and Ru K-edge EXAFS oscillation function (*k*^3^*χ*(*k*)) of the Pd–Ru, Pd and Ru samples, and it can be confirmed that the synthetic materials have the silmilar local structure as Ru metal. The obtained first nearest neighbour distances for each cation are summarized in Table 2. The Pd–Pd (2.740(1) Å) and Ru–Ru (2.679(1) Å) distances in both end-members are consistent with the published bond distances, respectively. The atomic radii of Pd and Ru are 1.37 Å and 1.34 Å, respectively. The Ru-(Ru,Pd) distances in an ideal 1 : 1 Pd–Ru solid-solution can be estimated as 34 + (1.37 + 1.34)/2 = 2.695 Å. The observed Pd-(Ru,Pd) (2.731(3) Å) and Ru-(Ru,Pd) (2.699(1) Å) distances were smaller and larger than Pd and Ru, respectively, in the Pd_0.5_Ru_0.5_ sample, which indicated that a solid-solution state is formed.

**Table 2:** Atomic distance by XAFS data

Sample	Pd-(Pd,Ru), (Å)	Ru-(Ru,Pd), (Å)
Pd	2.740(1)	—
Pd : Ru = 1 : 1	2.731(3)	2.699(1)
Ru	—	2.679(1)

### Atomic emission spectra

**Fig. 9:**
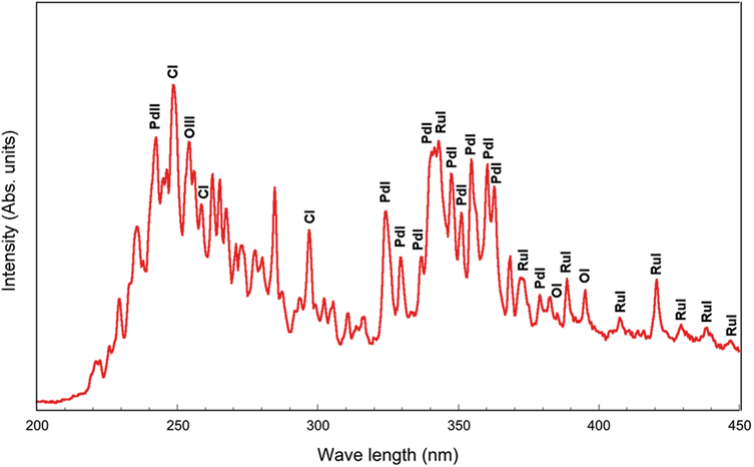
Atomic emission spectra collected from the plasma discharge zone.

Atomic emission spectra collected from the plasma discharge zone are given in Fig. 9. From the atomic emission spectrum, the peaks of Pd (PdI and PdII) and Ru (RuI) are identified, which showed that the Pd and Ru ions were generated by the pulsed discharges. The CI, OI and OIII peaks were also observed, which might have appeared due to the ethanol solution.

**Fig. 10:**
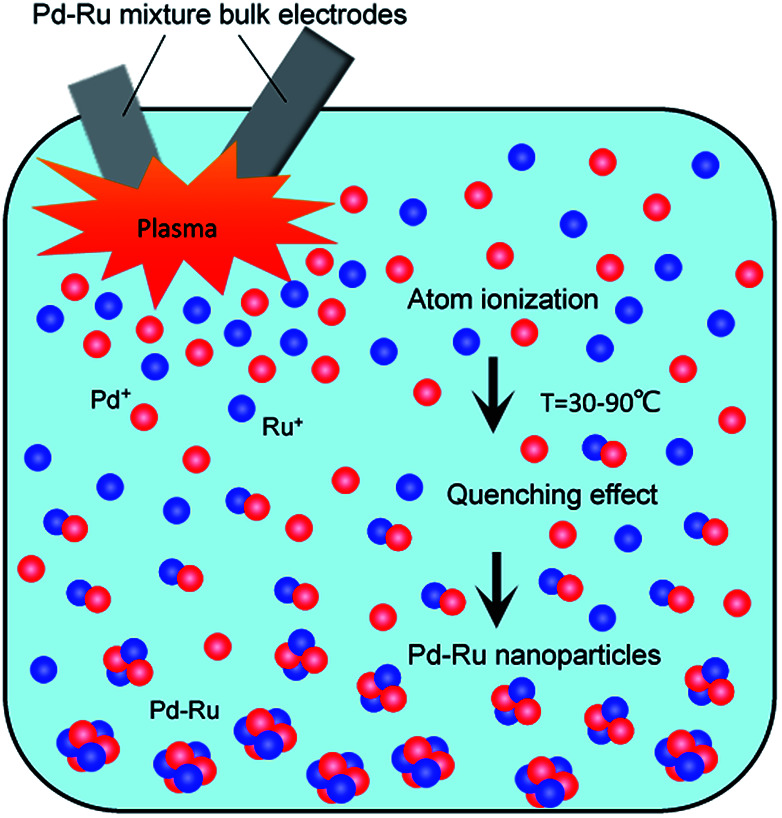
Illustration of the Pd–Ru alloy nanoparticle formation under pulsed plasma in liquid method.

Fig. 10 shows the illustration of the Pd–Ru alloy nanoparticle formation under pulsed plasma in liquid method. During spark discharge, Pd and Ru atoms were ionized by the high temperature of the pulsed plasma, and the Pd^3+^ and Ru^3+^ ions appeared. Under cooling, neighbouring Pd^3+^ and Ru^3+^ ions gather, and formed Pd–Ru solid-solution clusters. It is assumed that by the spark discharge of Pd–Ru alloy electrodes the Pd^3+^ and Ru^3+^ ions exist close to each other and easily react each other to form a cluster. As a result, the Pd–Ru solid-solution nanoparticles are succeeded to be produced there in spite of immiscible system. The short pulse duration and quenching effect also prevents particles size growth enable us to obtain the metastable solid-solution nanoparticles. However, the agglomeration of NPs occurs at a certain stage because of the small particle sizes and some other internal forces; therefore, slightly agglomeration of clusters was observed.

## Conclusions

We succeeded in synthesizing Pd–Ru series nanoparticles in solid solution with the Pd : Ru composition ratios of 1 : 3, 1 : 1 and 3 : 1 by the PPL method using Pd–Ru mixture bulk electrodes with the respective compositions. The resulting 1 : 1 and 3 : 1 Pd–Ru particles are in the FCC phase, while the 1 : 3 Pd–Ru particles are in the BCC phase. The lattice parameter increased with the increase in the Pd concentration and followed the Vegard's law. The EDX, XPS and XAFS results also confirmed the solid-solution state. The sizes of the solid-solution alloy particles were less than 10–20 nm. We expect the as-synthesized Pd–Ru solid-solution nanoparticles to show comparable or better catalytic properties than Rh or Pd nanoparticles.

## Conflicts of interest

There are no conflicts of interest to declare.

## Supplementary Material
